# Intracerebral Hemorrhage and Exposure to Antithrombotic Drugs

**DOI:** 10.1001/jamanetworkopen.2021.9175

**Published:** 2021-05-03

**Authors:** Peter Brønnum Nielsen, Truman J. Milling, Gregory Y. H. Lip

**Affiliations:** Aalborg Thrombosis Research Unit, Department of Clinical Medicine, Faculty of Health, Aalborg University, Aalborg, Denmark; Department of Cardiology, Aalborg University Hospital, Aalborg, Denmark; Seton Dell Medical School Stroke Institute, Neurology, Austin, Texas; Aalborg Thrombosis Research Unit, Department of Clinical Medicine, Faculty of Health, Aalborg University, Aalborg, Denmark; Liverpool Centre for Cardiovascular Science, University of Liverpool and Liverpool Heart and Chest Hospital, Liverpool, United Kingdom

Intracerebral hemorrhage (ICH) is a devastating clinical manifestation of bleeding into the brain parenchyma from a ruptured arterial vessel, and antithrombotics are known to be associated with worse ICH outcomes. Among patients with, for example, atrial fibrillation or venous thromboembolism, we accept this increased bleeding risk because the known reduction in thrombosis, such as stroke, far outweighs it. Despite the net benefit, during the warfarin era, only half of the patients who should have been undergoing anticoagulation actually were.^1^ Thus, we hoped that direct oral anticoagulants (DOACs), thought to be safer drugs might make inroads into this undertreated population. To achieve much greater stroke and other thrombosis prevention, we had to accept that risk of bleeding, including more ICH events, might worsen somewhat.

In this issue of *JAMA Network Open*, the findings of Hald et al^2^ are reassuring in this regard because they found that despite increased use of anticoagulants, largely driven by DOAC use, ICH prevalence has not increased in a Danish population. Hald et al^2^ investigated associations between exposure to antithrombotic drugs and subsequent odds of ICH among Danish residents. Using a case-control study design, they identified cases as individuals with an incident ICH diagnosis obtained from a dedicated stroke registry. The control population was sampled from Danish residents free of ICH, thus exploiting the strengths of risk set sampling, and controls were matched with cases by age, sex, and calendar year. Among 16765 case patients, incident ICH was significantly associated with antithrombotic use compared with matched controls. The strongest association was found among users of vitamin K antagonists (odds ratio [OR], 2.76; 95% CI, 2.58-2.96).

Epidemiologic studies^3,4^ have suggested that the strongest clinical risk factor associated with ICH is hypertension, but psychosocial factors and lifestyle (in particular alcohol abuse) have been reported to be more prevalent among patients who present with ICH. Although antithrombotic treatment is known to be associated with ICH, the medication itself is unlikely to be the cause of the ICH. However, concurrent antithrombotic treatment is a modifiable risk factor for hematoma expansion and thus crucial to identify early to mitigate poor outcomes. Indeed, ICH risk can be assessed using bleeding risk scores, such as the HAS-BLED score (hypertension, abnormal renal and liver function, stroke, bleeding, labile international normalized ratio, elderly, and drugs or alcohol), although such scores should be appropriately used to flag modifiable bleeding risk factors to mitigate harm and to schedule patients at high risk for bleeding for early review and follow-up.^5^ An integrated and holistic patient approach has been shown in the prospective mobile atrial fibrillation application II trial in which intervention with the ABC (Atrial fibrillation Better Care) pathway, with proactive use of the HAS-BLED score, was associated with less major bleeding and an increase in oral anticoagulation use compared with usual care.^6^

The provided evidence from Hald et al^2^ on the strength of the association between antithrombotic use and ICH carries little clinical value in terms of how best to approach patients who present with an ICH. We already know, for example, that patients with ICH and atrial fibrillation are at high risk for subsequent ischemic stroke and death, but the immediate concern is that of recurrent bleeding. Obviously, clinical factors determining the indication for antithrombotic use is of key importance because the treatment has been initiated to prevent ischemia, including ischemic stroke.

In confined analyses that focused on patients with atrial fibrillation or venous thromboembolism as an indication for antithrombotic treatment, the results generally reflected those of the main analyses. However, among dabigatran users, the risk of ICH was lower than among those who never used antithrombotic drugs (OR, 0.80; 95% CI, 0.56-1.13). Clearly, this is counterintuitive despite an expected class effect of DOACs having a relative low risk of ICH compared with warfarin.^7^ A similar and potentially spurious association was observed when using warfarin as the reference group to compare the risk of ICH with the use of rivaroxaban (OR, 1.20; 95% CI, 1.03-1.41), which again challenges the expected class effect of DOACs with the lower risk of ICH compared with warfarin.^7^ These results highlight that the evidence provided should not be used to guide clinical practice.

Nevertheless, the study presents a compelling overview of the landscape of ICH during the past few decades and confirms that antithrombotic use has a rare but potentially devastating adverse effect. However, despite increased use, particularly of DOACs, the incidence of ICH has not increased, suggesting that a net benefit is maintained at a population level. Prospective randomized clinical trials are needed to better define and expand the patient population that may benefit from anticoagulation, including those reinitiating anticoagulation who sustain an ICH. Of note, nontraumatic ICH may differ from traumatic ICH in the risk of subsequent ischemic and bleeding events; hence, a specific trial on restarting anticoagulation after traumatic ICH is planned.^8^ Nontraumatic ICH is more complicated, depending on site of the bleed and other imaging features, such as the presence of multiple microbleeds. Patients with nontraumatic ICH require an integrated care approach, including input from stroke neurologists, cardiologists, neurosurgeons, primary care physicians, and the patient.
